# PD-1/PD-L1 immune checkpoint inhibitors in metastatic triple-negative breast cancer: a systematic review and meta-analysis

**DOI:** 10.3389/fimmu.2023.1206689

**Published:** 2023-06-12

**Authors:** Yinan Yu, Xueying Jin, Xiaoling Zhu, Yan Xu, Wei Si, Jianguo Zhao

**Affiliations:** Department of Oncology, Shaoxing People’s Hospital, Shaoxing, Zhejiang, China

**Keywords:** chemotherapy, immune checkpoint inhibitors, meta-analysis, triple negative breast cancer, systematic review

## Abstract

**Background:**

PD-1/PD-L1 immune checkpoint inhibitors (ICIs) have been controversial in the treatment of metastatic triple negative breast cancer (mTNBC). We collected randomized controlled trials in accordance with the study and carried out meta-analysis to comprehensively evaluate the efficacy and safety of immune checkpoint inhibitors in mTNBC.

**Aim:**

To systematically evaluate the efficacy and safety of PD-1/PD-L1 ICIs (hereinafter referred to as ICIs) in the treatment of mTNBC.

**Methods:**

As of 2023.2.5, Medline, PubMed, Embase, Cochrane library database and Web of Science were searched to determine the study in accordance with the trial of ICIs in the treatment of mTNBC. The assessment endpoints included objective response rate (ORR), progression-free survival (PFS), overall survival (OS), and safety. Meta-analysis of the included studies was performed using Revman 5.4.

**Results:**

A total of six trials with 3172 patients were included in this meta-analysis. The ORR of ICIs combined with chemotherapy was significantly improved compared with chemotherapy (HR=0.88, 95%CI: 0.81-0.94, I^2 ^= 0%). For PFS, the experimental group were better than the control group in both intention-to-treat (ITT) population and PD-L1 positive population, showing statistical significance (ITT: HR=0.81, 95%CI: 0.74-0.89, P<0.05, I^2 ^= 0%; PD-L1 positive: HR=0.72, 95%CI: 0.63-0.82, P<0.05, I^2 ^= 18%); For OS, in the ITT population, no statistical difference was observed in either ICIs combined with chemotherapy(HR=0.92, 95%CI: 0.83-1.02, P=0.10)or immune monotherapy(HR=0.78, 95%CI: 0.44-1.36, P=0.37), in the PD-L1 positive population, ICIs group had better OS than chemotherapy group (HR=0.83, 95%CI: 0.74-0.93, P < 0.05); In safety, serious adverse event (SAE) was no statistically significant difference between the ICIs group and the chemotherapy group; however, the incidence of immune-related adverse event (irAE) was significantly higher in the ICIs group than in the chemotherapy group (HR=2.15, 95%CI: 1.45-3.19, P < 0.05).

**Conclusion:**

ICIs combined with chemotherapy significantly improved the PFS of mTNBC, however, ICIs only improved the OS in PD-L1 positive people, and no statistical difference was observed in ITT population; while benefiting from ICIs, we found that irAE in ICIs group increased significantly, and its high rate of adverse events still needs to be taken seriously.

## 1 Introduction

Breast cancer is the most common malignant tumor among women worldwide. According to the data of the International Agency for Research on Cancer, there will be 2.26 million new cases of breast cancer worldwide in 2020, and nearly 700,000 deaths from breast cancer (1). As a special subtype of breast cancer, TNBC accounts for 15%-20% of all breast cancers (2), It is characterized by high invasiveness, easy recurrence and metastasis (3), and poor prognosis.Meanwhile, due to the molecular characteristics of TNBC, it is not sensitive to traditional endocrine therapy and targeted therapy (4), chemotherapy is still the only treatment option (5). However, only one-third of patients responded to first-line chemotherapy, and the benefits were limited (median OS was less than 2 years) (6, 7).

In recent years, immuno checkpoint inhibitors, as a new type of immunotherapy, have made important progress in a variety of solid tumors. Programmed death protein-1 (PD-1) and its ligand (PD-L1) inhibitors are immune sentinel monoclonal antibodies that reactivates the body’s immune system to defend against cancer cells by blocking the PD-1/PD-L1 signaling pathway (8), compared with other subtypes, TNBC has higher tumor mutation burden, higher PD-L1 expression level, and more immune cell infiltration in the tumor microenvironment. However, with the results of IMpassione130, IMpassion131 and other studies reported, whether ICIs monotherapy or combination chemotherapy can improve efficacy in the treatment of TNBC is controversial.

In order to further understand the clinical efficacy of ICIs in patients with mTNBC, we conducted a meta-analysis of all RCT studies in patients with mTNBC receiving ICIs, and comprehensively evaluated the efficacy and safety of ICIs in the treatment of mTNBC.

## 2 Methods

### 2.1 Data sources and search strategy

Relevant studies were sourced from Medline, PubMed, Embase, Cochrane library, Web of Science; the search strategy employed relevant keywords and medical subject heading (MeSH) terms, including the following: [(triple-negative breast cancer or triple negative breast neoplasm or TNBC or triple-negative breast carcinoma)] AND (Immune checkpoint inhibitors or Immune Checkpoint Blockers or PD-L1 Inhibitor or PD-1 Inhibitor or pembrolizumab or atezolizumab or nivolumab or durvalumab or camrelizumab or sintilimab).

### 2.2 Inclusion and exclusion criteria

This meta-analysis followed the PRISMA (Preferred Reporting Items for Systematic Reviews and Meta-analyses) guidelines (9).

#### 2.2.1 Inclusion criteria

1. Type of study: Prospective randomized controlled trial; 2. Subjects: Patients with a definite pathological diagnosis of triple-negative breast cancer, including PD-L1 assessment; 3. Intervention measures: The experimental group was treated with ICIs plus chemotherapy or immune monotherapy, while the control group was treated with chemotherapy(no chemotherapy regimen); 4. Outcome measures: Hazard ratio (HRs) and 95% confidence interval (CI) for ORR, PFS, OS, and incidence of adverse reactions.

#### 2.2.2 Exclusion criteria

1. non-RCT studies such as single-arm trials and retrospective studies, animal trial, case reports, meta-analysis and observational retrospective study; 2. Literature for which data cannot be extracted directly or indirectly; 3. Non-English language literature.

### 2.3 Quality assessment and data extraction

The risk of bias was discussed and assessed according to the Cochrane Collaboration’s Risk of Bias tool by two independent investigators (Xueying Jin and Yan Xu), the risk of bias of the included literature was assessed in terms of the following six dimensions: random sequence generation (selection bias), allocation concealment (selection bias), blinding of participants and personnel (performance bias), blinding of outcome assessment (detection bias), incomplete outcome data (attrition bias), and selective reporting (reporting bias), and was categorized into three types: low risk, high risk, and uncertain risk, data extraction was conducted by mutual agreement and all potential disagreements were resolved by consensus (10).

Two investigators (Yinan Yu and Wei Si) extracted the following information independently: name of the studies, first author, publication year, study design, study phase, lines of treatment, participant characteristics (quantity etc.), study sample size, PD-L1-positive definition assays, intention-to-treat population, PD-L1 status subgroups, HRs and 95% CI for ORR, PFS and OS, and the number of SAEs and irAE. When multiple results are reported, only the latest results are used. All disagreements were resolved by a third investigator.

### 2.4 Outcome definitions

The primary endpoint was PFS and OS, the PFS is defined as the time from randomization to the time of radiographic progression (as assessed by iRECIST 1.1) or death from any cause during the study, the OS will be calculated from the time of randomization until death.

The secondary endpoint was ORR, the ORR is defined as the proportion of patients with tumor shrinkage of a certain amount and maintained for a certain period of time, including complete response (CR) and partial response (PR) cases.

Safety endpoints included the incidence of all serious adverse events (SAEs; According to the WHO Toxicity Standards or the National Cancer Institute Standard Classification of Adverse Events Generic Terms) and immune-related adverse events.

### 2.5 Statistical analysis

Statistical analysis was performed using Revman version 5.4 by an independent statistician (Yinan Yu), and the generic inverse variance was selected for the PFS and OS hazard ratio (HR) data to compute and record the log [HR] and the corresponding SE, dichotomous data types were selected for data such as ORR and adverse events. The heterogeneity of the studies was estimated using the I^2^ statistics (11), and random-effect models were used for P<0.1 or I^2^>50%; otherwise, the fixed-effects model was used. Statistical significance was set at P < 0.05.

## 3 Results

### 3.1 Study and data selection

**Figure 1:**
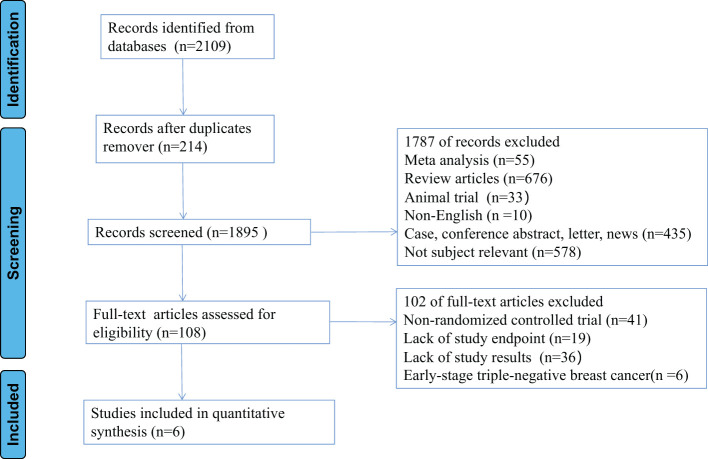
Flow diagram of study selection process.

The flowchart of the detailed search process is illustrated in **Figure 1**. A total of 2109 studies were retrieved through the primary search strategy, of which 214 were duplicates. Of the remaining 1895 studies, 676 review articles, 55 meta-analysis, 33 animal trial, 10 non-English, 578 not subject relevant, 435 conference abstracts, letters, news and case reports were excluded. Furthermore, 102 studies were excluded following a detailed evaluation of the full text for the following reasons: A total of 41 were non-randomized controlled trial, 55 lacked study endpoints and results, and 6 Early-stage triple-negative breast cancer. Consequently, 6 clinical randomized controlled trials with 3172 patients were enrolled in the current meta‐analysis.

### 3.2 Study characteristics and quality assessment

The characteristics of the included trials are shown in **Table 1**. A total of six trials with 3172 mTNBC patients were included,1761 of whom had PD-L1 positive mTNBC. Four of these studies[IMpassion130 (12), IMpassion131 (13), KEYNOTE355 (14), ALICE (15)] compared the clinical outcomes following immune combined with chemotherapy and chemotherapy, whereas the other 2 studies [SAFIR02-BREAST (16), KEYNOTE119 (17)] compared the clinical outcomes following Immune monotherapy and chemotherapy. Of the 6 included studies,5 included all-subject ORR outcomes, 4 included all-subject PFS outcomes, 3 included PD-L1 positive subject PFS outcomes, 5 included all-subject OS outcomes, 5 included PD-L1 positive subject PFS outcomes, and 5 reported the incidence of SAE or irAE in subjects.

**Table 1:** Baseline characteristics of included study.

Authors	Study name (phase/year)	Key inclusion criteria	Population characteristics	treatment group	control group	Number of patients with mTNBC, N	PD-L1-positive subset, N	outcomes
Schmid et al	IMpassion 130 (phase III, 2021)	18 years of age or older. ECOG 0-1. Previously untreated locally recurrent inoperable or mTNBC. DFI >12 months(including taxanes).	24.3% with age > 65; 67.5% of white race; >3 metastatic sites 25.1%; liver metastases 27.1%; 51.1% with prior taxane.	Atezolizumab+Nab-paclitaxel	Placebo+Nab-paclitaxel	902 (1:1)	369 (185:184)	ORR,PFS,OS,SAE,irAE
Miles et al	IMpassion 131 (phase III,2021)	ECOG 0-1.eligible for taxane therapy. Previously untreated locally recurrent inoperable or mTNBC. DFI ≥12 months.	Median age 54; 57.5% of white race; >3 metastatic sites 23.5%, liver metastases 27.5%; 48.4% with prior taxane.	Atezolizumab+paclitaxel	Placebo+paclitaxel	651 (2:1)	292 (191:101)	ORR,PFS,OS,SAE,irAE
Cortes et al	KEYNOTE355 (phase III,2022)	18 years of age or older. ECOG 0-1. Previously untreated locally recurrent inoperable or mTNBC. DFI >6 months.	21.3% with age >65; 68.4% of white race; ≥3 metastatic sites 43.1%; liver metastases 29.4%; 52.7% with prior taxane.	Pembrolizumab+chemotherapy	Placebo+chemotherapy	847 (2:1)	636 (425:211)	ORR,PFS,OS,SAE,irAE
Kyte et al	ALICE (phase II,2022)	18 years of age or older. ECOG 0-1. metastatic TNBC. DFI ≥12 months.	Median age 58; >3 metastatic sites 14.7%; liver metastases 36.8%;	Atezolizumab+Chemotherapy	Placebo+Chemotherapy	68 (3:2)	27 (19:8)	ORR,PFS(ITT only),SAE,irAE
Bachelot et al	SAFIR02-BREAST (phase II,2021)	ECOG 0-1. metastatic TNBC.	—	Durvalumab	chemotherapy	82 (47:35)	32(18:14)	OS
Winer et al	KEYNOTE119 (phase III,2021)	18 years of age or older. ECOG 0-1. metastatic TNBC. previous treatment with an anthracycline or a taxane.	15.8% with age ≥65; history of brain metastases 6.8%; liver metastases 29.4%; 79.1% with Prior neoadjuvant or adjuvant therapy.	Pembrolizumab	investigator-choice chemotherapy	622 (1:1)	405 (203:202)	ORR,OS,SAE,irAE

IMpassion 130, IMpassion 131, KEYNOTE 355, ALICE, KEYTONE119 reported the results of the ORR; IMpassion 130, IMpassion 131, KEYNOTE 355, ALICE reported the results of the PFS (IMpassion 130, IMpassion 131, KEYNOTE 355 reported the results of the PFS in PD-L1 positive population); IMpassion 130, IMpassion 131, KEYNOTE 355, SAFIR02-BREAST, KEYTONE119 reported the results of the OS (including PD-L1 positive population); IMpassion 130, IMpassion 131, KEYNOTE 355, ALICE, KEYTONE119 reported the results of the SAE and irAE. DFI: disease-free interval.

**Figure 2:**
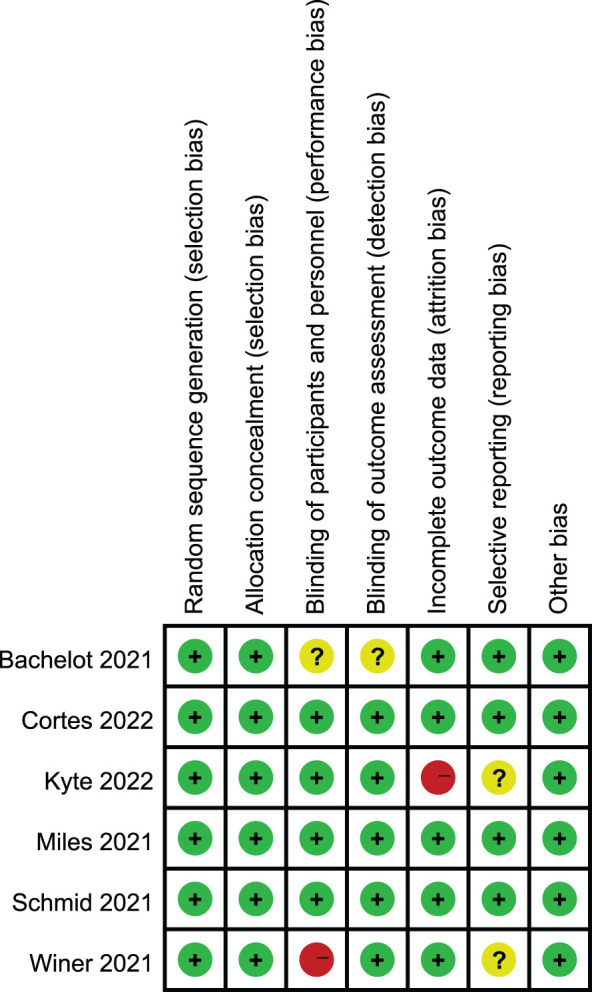
Risk of bias summary: review for the enrolled study. **+**: low risk, **-**:high risk,**?**: unclear risk.

The results of the Cochrane risk bias assessment are shown in **Figure 2**. All six included studies were of high quality and mostly at low risk of bias (green section in the figure). Keynote119 (winer 2021) had performance bias, ALICE (kyte 2022) had attrition bias due to having patients lose follow-up, and SAFIR02-BREAST (Bachelot 2021) had unclear risk of double blindness because it was not indicated in the study design.

### 3.3 Antitumor efficacy outcomes

#### 3.3.1 ORR of the ITT population

**Figure 3:**
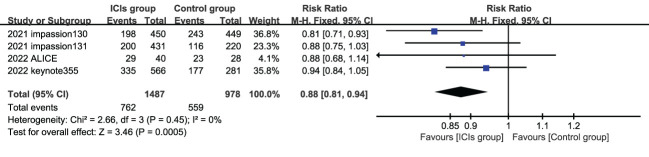
Forest plots of ORR associated with ICIs (ICIs ± chemotherapy) versus chemotherapy. ORR, objective response rate; ICIs, immune checkpoint inhibitors.

5 studies reported the subjects ORR, and the heterogeneity test indicated high heterogeneity, the labbe chart showed that KEYNOTE119 deviated from the distribution, and no heterogeneity was found after removing this article (I^2 ^= 0). Therefore, a summary analysis of ORR was finally conducted for 4 studies (4 experimental groups were all ICIs combined with chemotherapy) Compared with control group, ORR in experimental group was significantly improved (HR=0.88, 95%CI 0.81-0.94, P<0.05) (**Figure 3**). KEYNOTE119 showed that pabolizumab alone did not improve ORR in mTNBC.

#### 3.3.2 PFS of the ITT population and PD-L1 positive population

**Figure 4:**
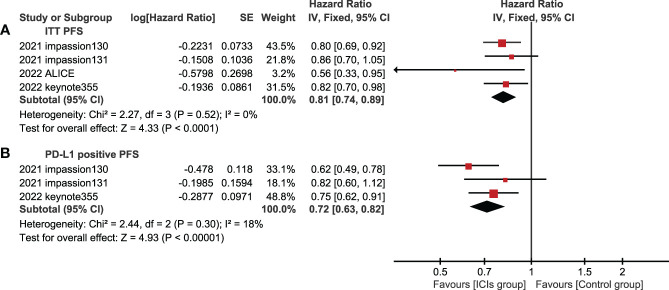
Forest plots of PFS associated with ICIs+chemotherapy versus chemotherapy. **(A)**, ITT population; **(B)**, PD-L1 positive population; PFS, progression-free survival; ICIs, immune checkpoint inhibitors.

4 studies (experimental groups were all ICIs combined with chemotherapy) reported PFS in ITT populations, three of which reported PFS in PD-L1 positive population. In both ITT population and PD-L1 positive population, PFS in the experimental group were better than those in the control group, showing statistical significance(ITT: HR=0.81, 95%CI: 0.74-0.89, P<0.05, I^2 ^= 0%; PD-L1 positive: HR=0.72, 95%CI: 0.63-0.82, P<0.05, I^2 ^= 18%) (**Figure 4**).

#### 3.3.3 OS of the ITT population and PD-L1 positive population

**Figure 5:**
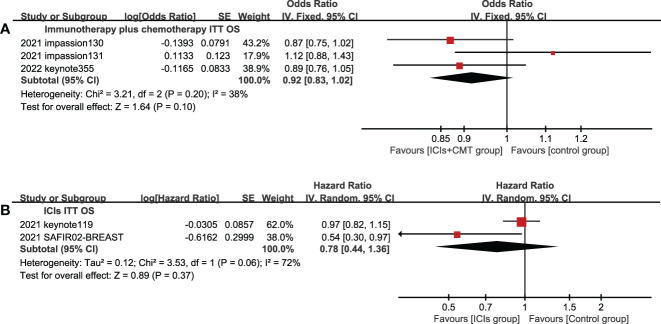
Forest plots of OS associated with ICIs ± chemotherapy versus chemotherapy. **(A)**, ICIs+chemotherapy group; **(B)**, ICIs group; OS, overall survival; ICIs, immune checkpoint inhibitors; CMT, chemotherapy.

**Figure 6:**
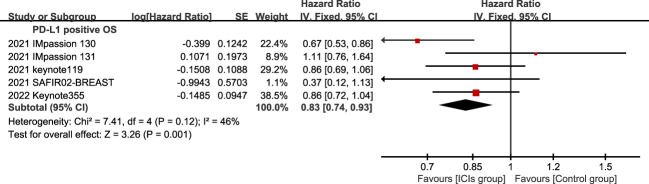
Forest plots of OS associated with ICIs (ICIs ± chemotherapy) versus chemotherapy. PD-L1 positive population; OS, overall survival; ICIs, immune checkpoint inhibitors.

5 studies reported OS in the ITT population and PD-L1 positive population. In the ITT population, according to the treatment plan of the experimental group, ICI combined chemotherapy (P=0.10) and ICIs single drug (P=0.37) were respectively analyzed,there was no statistical difference in OS between the experimental group and the control group (**Figure 5**), and ICIs failed to improve the mTNBC OS. In the PD-L1 positive population, the OS of the experimental group was significantly better than that of the control group, and the difference was statistically significant (HR=0.83, 95%CI: 0.74-0.93, P<0.05, I^2 ^= 46%) (**Figure 6**).

### 3.4 Safety outcomes

**Figure 7:**
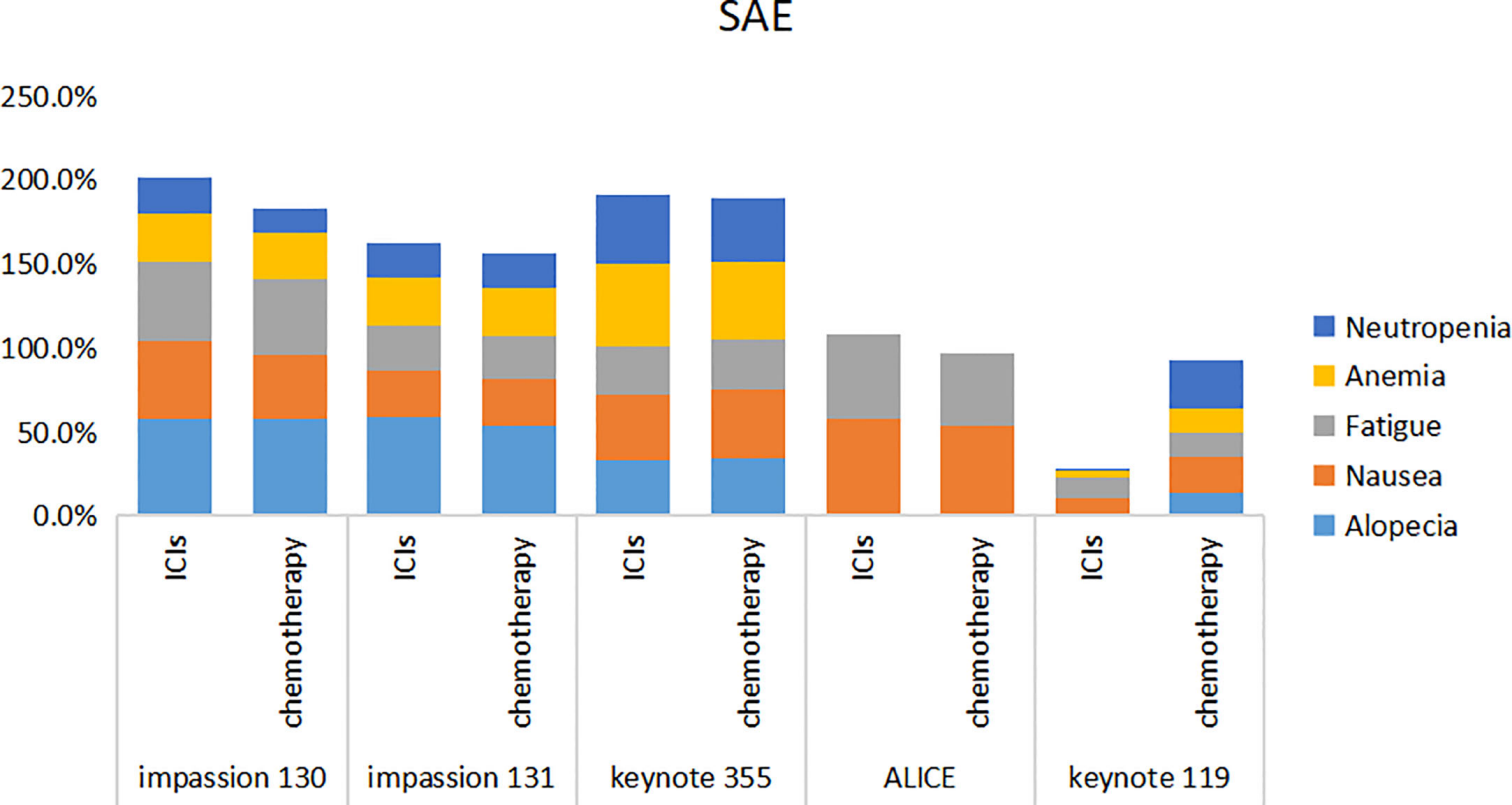
Stacked bar chart of SAE. SAE, serious adverse event; ICIs, immune checkpoint inhibitors.

**Figure 8:**
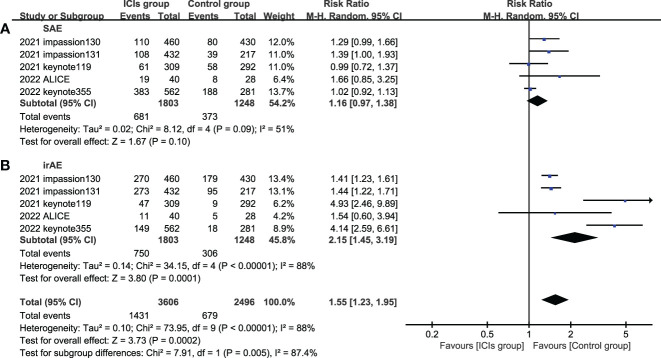
Forest plots of adverse event associated with ICIs (ICIs ± chemotherapy) versus chemotherapy. **(A)**, SAE, serious adverse event; **(B)**, irAE, immune-related adverse event; ICIs, immune checkpoint inhibitors.

**Figure 9:**
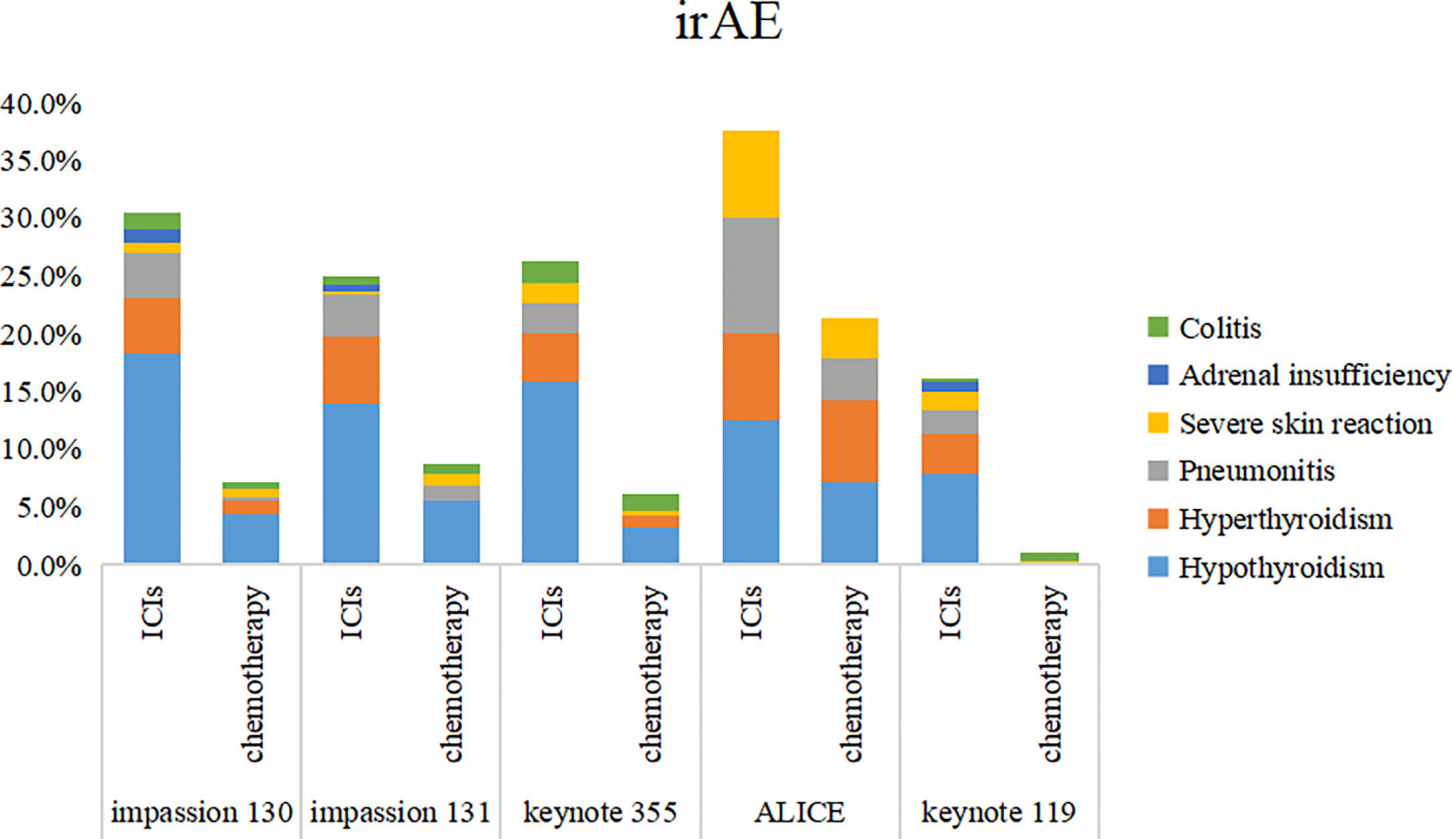
Stacked bar chart of irAE. irAE, immune-related adverse event; ICIs, immune checkpoint inhibitors.

5 studies included SAE and irAE during treatment, the intra-group heterogeneity test I^2^ of both groups was greater than 50%, so random effects model was used for analysis. For adverse events, SAE showed no significant difference between the experimental group and the control group (HR=1.16, 95%CI: 0.97-1.38, P=0.09), there are various SAE reports, among which alopecia,nausea,fatigue,anemia and neutropenia are common (**Figure 7**), while the incidence of treatment-related irAE in the experimental group was significantly higher than that in the control group (HR=2.15, 95%CI: 1.45-3.19, P<0.05) (**Figure 8**), most common irAE was hypothyroidism (**Figure 9**).

## 4 Discussion

TNBC is the most difficult subtype of breast cancer to treat due to the lack of therapeutic intervention targets (18, 19), chemotherapy is still the standard systemic treatment for most patients, but the rapid development of drug resistance after chemotherapy is inevitable (20). In recent years, the emergence of immune checkpoint inhibitors has brought a new dawn to TNBC patients, TNBC has a high tumor mutation burden and abundant lymphocyte infiltration around the tumor, which provides an antigenic basis for the recognition of immune cells and a good immune microenvironment basis for the application of ICIs (21, 22). However, with the reported results of several studies, there is still no conclusion on whether ICIs can improve the efficacy of mTNBC.

This meta-analysis showed significant improvements in ORR and PFS in combination with ICIs compared with chemotherapy alone (differences in PFS between immunomonotherapy and chemotherapy were not compared as PFS were not reported in either of the two included immunomonotherapy studies, KEYNOTE119 reported that the ORR of immune monotherapy and chemotherapy was 30% and 33%, respectively,failed to improve the ORR), for OS, no statistical difference was found in ITT population, no matter ICIs combined chemotherapy or immunomonotherapy, compared with chemotherapy alone, however, significant improvement was still observed for PD-L1 positive patients. These RCTS showed that for overall mTNBC patients, the addition of ICIs to chemotherapy could significantly improve the short-term efficacy, however, no significant benefit was seen for OS, further subgroup analysis showed that for mTNBC patients with PD-L1 positive, ICIs improved ORR, PFS and OS. This is undoubtedly a promising choice for clinical application. On the other hand, it also proves that the efficacy of ICIs is closely related to the expression of PD-L1. In addition, our summary found that the incidence of irAE in the ICIs group was significantly higher than that in the chemotherapy group, indicating that while ICIs increased the efficacy benefits, they also significantly increased the risk of irAE in patients, which also warned us that in clinical application, patients’ status still needs to be evaluated, pros and cons weighed, and appropriate treatment methods should be selected.

In general, although ICIs combined with chemotherapy and immunomonotherapy failed to improve the OS of ITT patients, the short-term efficacy of ICIs combined with chemotherapy was satisfactory. Based on the positive results of IMpassion130 and KEYNOTE355 and the negative results of KEYNOTE119, ICIs combined with chemotherapy was still more dominant, the two complement each other and have synergistic effects, mainly considering the following aspects: 1. after killing tumor cells, chemotherapy can more fully expose tumor antigens, providing the antigenic basis for the application of ICIs; 2. chemotherapy can reduce the TMB of patients in a short time, providing a time buffer for ICIs to take effect, and at the same time can carry out intensive siege therapy on single tumor cells; 3. Studies (23) have shown that immunotherapy can weaken basal layer cell-mediated chemotherapy resistance and restore tumor response rate to chemotherapy through effector T cells in tumor microenvironment.

This study also has the following limitations: First, ICIs used to treat mTNBC patients are different, and there is a mild and moderate heterogeneity in PFS and OS studies, respectively. Second, due to the small number of RCTS included in this study, it is not enough to conduct publication bias assessment, and there may be potential publication bias. Third, various studies combined with chemotherapy regimen and chemotherapy regimen of control group are not completely consistent, which may lead to bias in results. Fourthly, the detection methods of PD-L1 in various studies are different, IMpassion 130, IMpassion 131, SAFIR02-BREAST were assessed by VENTANA PD-L1 (SP142) immunohistochemical testing, while KEYNOTE 355 and KEYNOTE 119 were assessed using the IHC 22C3 pharmDx assay, although the data included in this study showed that PD-L1 positive population had benefits in both PFS and OS in the ICIs group, this is still a heterogeneity that needs to be carefully addressed in future evaluation.

In conclusion, although the FDA withdrew Atezolizumab in combination with albumin-bound paclitaxel (Abraxane) chemotherapy for the treatment of patients with mTNBC whose tumors express PD-L1 due to the difference between IMpassion130 and IMpassion131, the application of ICIs in patients with tolerable mTNBC, especially in PD-L1 positive population, is still worthy of expectation.

## 5 Conclusion

The current analysis suggests that ICIs combined with chemotherapy significantly improves the near-term efficacy of mTNBC, however, whether ICIs is combined with chemotherapy or ICIs monotherapy, ICIs only improved the OS in PD-L1 positive people, and no statistical difference was observed in ITT population. Of course, with the benefit of ICIs, the incidence of irAE is also significantly increased, which is also a problem that we need to carefully consider in future treatment options.

## Data availability statement

The original contributions presented in the study are included in the article/supplementary material. Further inquiries can be directed to the corresponding author.

## Author contributions

XZ introduced the status quo of triple negative breast cancer treatment, WS searched RCT studies according to the requirements, XJ and YX conducted literature quality assessment, YY collated and analyzed all the data, and was a major contributor in writing the manuscript. All authors contributed to the article and approved the submitted version.
